# Association of Follicle-Stimulating Hormone Receptor Polymorphisms with Ovarian Response in Chinese Women: A Prospective Clinical Study

**DOI:** 10.1371/journal.pone.0078138

**Published:** 2013-10-22

**Authors:** Yuanliang Yan, Zhicheng Gong, Lu Zhang, Yanping Li, Xiong Li, Lin Zhu, Lunquan Sun

**Affiliations:** 1 Department of Pharmacy, Xiangya Hospital, Central South University, Changsha, Hunan, P. R. China; 2 School of Pharmaceutical Science, Central South University, Changsha, Hunan, P. R. China; 3 Center for Molecular Medicine, Xiangya Hospital, Central South University, Changsha, Hunan, P. R. China; 4 Reproductive Medicine Department, Xiangya Hospital, Central South University, Changsha, Hunan, P. R. China; University Hospital of Münster, Germany

## Abstract

**Background:**

Follicular stimulating hormone (FSH) is a glycoprotein and widely used for the treatment of infertility; FSH action is mediated by FSH receptor (FSHR), SNPs of which determine the ovarian response. Two polymorphisms of the FSHR gene were identified, which caused a change of threonine (T) to alanine (A) at position 307 and asparagine (N) to serine(S) at position 680. Both polymorphic sites give rise to three discrete variants of the FSHR: TT, TA, and AA for position 307; NN, NS, and SS for position 680.

**Methodology/Principal Findings:**

450 Chinese women were recruited in an assisted reproductive technology program from October 2011 to March 2012. FSHR polymorphisms at the positions 307 and 680 were examined by PCR-RFLP. Serum FSH and estradiol level, FSH amount, ovarian response and pregnancy rate were recorded during treatment. The basal FSH levels were higher in AA [7.38 ± 2.07 vs 6.34 ± 1.75, 6.63 ± 1.94, P<0.05, 95% CI (6.75, 8.01)] and SS [7.51 ± 2.01 vs 6.31 ± 1.75, 6.66 ± 1.96, P<0.05, 95% CI (6.88, 8.15)] compared to other genotypes respectively; the days for ovulation induction was slightly longer in AA and SS. Women with AA and SS have higher rates of poor response compared to carriers of other genotypes (P<0.05). Furthermore, there is a nearly complete linkage between these two polymorphisms in Chinese women (D’=0.95, r^2^=0.84).

**Conclusions/Significance:**

In Chinese women receiving ART, the subjects with AA and SS genotypes have higher basal FSH levels, and these genotypes are associated with an increased risk of poor response. Our data suggested that the personalized FSH therapy may be applied according to patient’s genetic background in clinical settings. The linkage suggested that the polymorphisms of Thr307Ala and Asn680Ser may be used as TAG-SNP markers for analysis of potential genotyping in ART.

## Introduction

In assisted reproductive technology (ART) programs, the response of ovulating women to exogenous follicular stimulating hormone (FSH) is quite variable in individuals. Past clinical results indicated that it is difficult to prognosticate the ovarian response to intense gonadotropin stimulation. Poor ovarian response results in the insufficient stimulation and cycle cancellation [1]. Reversely, hyper-response will potentially induce serious and life threatening complication of ovarian hyper stimulation syndrome (OHSS). Cycle cancellation may be required to avoid the risk of ovarian enlargement and abdominal fluid extravasation [2]. Several parameters strived to assess ovarian reserve have been postulated as predictors of ovarian response [3]. The FSH level on day 3 of menstrual cycle seems to be one of the best predictive markers for ovarian reserve; however, the genetic background of individuals seems to determine the response of patients to FSH stimulation rather than the stimulation scheme [4].

FSH and FSH receptor (FSHR) play the essential roles in the regulation of steroidogenesis and follicle proliferation in ovary stimulation [5,6]. The FSHR is a trans-membrane protein consisting of intracellular, transmembrane and extracellular domains in granulosa cells [7-9]. FSHR is synthesized by a single-copy gene located in the region 2p21-16 [10]. The FSHR gene consists of 10 exons, in which the exon 10 encodes the C-terminal part of the extracellular domain, the transmembrane domain and the intracellular domain [11,12]. 

The gene of FSHR harbors more than 900 SNPs, two of which at positions of 307 and 680 are located in the exon 10 [13]. These SNPs cause a change of threonine to alanine at position 307 (Thr307Ala) and asparagine to serine at position 680 (Asn680Ser) [14]. Most studies focused on position 680 which is located in the intracellular region, while polymorphism at position 307 was rarely investigated. Position 307 codifies for an amino-acid located within the transmembrane region of FSHR protein [15]. This position is critical as it may be involved in the hormone-binding ability of FSHR and FSH-mediated signal transduction in the infertile women undergoing ovarian stimulation [16,17]. 

In the study of position 680 in women undergoing ovarian stimulation, the results demonstrated that carriers of SS variant had significantly higher basal FSH levels, and required higher doses of exogenous FSH for stimulation [18,19]. In another study, it was observed that the SS group showed the lowest estradiol levels at the time of human chorionic gonadotropin (hCG) injection after gonadotropin administration in Japanese women, but such a trait was not observed in German women [4,20]. However, the genotype at position 680 was not a predictor for OHSS development, but could correlate to the severity of symptoms of OHSS patients [21]. Thus, in a population of infertile women, FSHR gene polymorphism at position 680 is associated with the ovarian response to the controlled ovary hyperstimulation( COH). For the position 307, the subjects with AA genotype appeared to need a low amount of FSH for ovarian stimulation, and have an increased risk of developing OHSS in Indian women [22]. However, due to the small size of the study population in this study, the relationship between these polymorphisms and OHSS remains to be confirmed. Additionally, a strong linkage has been reported between these two polymorphisms, therefore, only one position needs to be tested as a TAG SNP [23-26]. However, to the best of our knowledge, no study has been conducted with respect to the association between the polymorphisms at positions 307 and 680 of FSHR and ART response in Chinese population. 

Here, we hypothesize that there is an association between FSHR polymorphisms (Thr307Ala and Asn680Ser) and ovarian response in Chinese women. To test this hypothesis, we employed the methods of Polymerase Chain Reaction-restriction polymorphism length fragment (PCR-RFLP) and direct sequencing to analyze the polymorphisms of FSHR gene at position 307 and 680 from 450 patients in Chinese population. The study demonstrated that the polymorphisms at positions 307 and 680 are associated with the ovarian response to FSH. Furthermore, the SNP at position 680 (Asn680Ser) was closely linked to the disequilibrium of Thr307Ala.

## Materials and Methods

### Subjects

The study protocol was approved by the Chinese clinical trial register (ChiCTR-OCH-12002155) and the research ethics committee, xiangya hospital, China. All subjects were women born in southern China. Peripheral blood was obtained from 450 females who are infertile and received *in vitro* fertilization (IVF) at the Reproductive Medicine Department of XiangYa Hospital from October 2011 to March 2012. The reason of infertility has been identified as male causes, tubal factor, or both. Male infertility refers to the inability of a couple to achieve a pregnancy after one year of unprotected sexual intercourse with a fertile female. Tubal infertility includes either tubal occlusion or adhesions. Mix factor infertility includes both male infertility and tubal infertility. Each patient received a full explanation of purpose of the study, and signed the consent form. All patients had body mass index of 16–35 kg/m^2^ with ages ranged from 20 to 46 years old.

### Treatment

Ovulation was induced according to the standard protocol using gonadotrophin-releasing hormone agonist. Ovarian was exposed to an empirical dose of Urofollitropin (Lizhu Pharmaceuticals, China), depending on serum estradiol levels and ultrasonography in order to reach an optimal ovarian response. Follicular development was monitored by transvaginal sonography. The monitoring started at 3rd or 4th day post stimulation and continued every other day. Sonography was performed daily when the leading follicle exceeded 17-18 mm in diameter. Ovulation was induced by 10,000 IU hCG when at least one follicle attained more than 20mm in diameter. Oocytes were collected by transvaginal ultrasoundguided aspiration 36h after hCG administration. 

 Basal levels of FSH, luteinizing hormone (LH) and estradiol on day 3 of the menstrual cycle were measured in one of the cycles before ovarian stimulation. In all subjects, the level of gonadotropins, number of follicles, number of retrieved oocytes, clinical pregnancy rate and the related parameters were recorded. The ovarian response was classified to poor (less than five), normal (between five and fourteen) and high (more than fourteen) based on the number of retrieved oocytes [27].. 

### DNA isolation and analysis of FSHR gene polymorphism

A volume of 1 ml whole blood was obtained from each subject, and was anticoagulated with EDTA. Genomic DNA was extracted from leukocytes in peripheral blood with the Wizard® Genomic DNA Purification Kit (Promega, USA) according to the manufacturers’ manual.

 For testing FSHR polymorphisms, the PCR reactions were performed in an Eppendorf Personal Cycler Thermocycler (Eppendorf, Germany) with 5ul DreamTaq TM Green PCR Master Mix (Fermentas, USA), 20 pmols of each primer and 100 ng of DNA in a final volume of 10 μL.

### PCR-RFLP analysis of the T307A variant

The polymorphism of T307A variant was detected by the nested PCR–RFLP method. Firstly, a 657 bp fragment of FSHR gene was amplified from the genomic DNA by PCR, PCR was ran at an annealing temperature of 58°C with 35 cycles of 95°C for 30 seconds, 58°C for 30 seconds, and 72°C for 45 seconds. The primers used in the reaction are upstream 5’TCTGAGCTTCATCCAATTTGCA3’, downstream 5’GGGAAAGAGGGCAGCTGCAA3’. The amplified fragment after the first round of PCR (2 μL) was further amplified by secondary PCR using another set of primers (upstream 5’CAAATCTATTTTAAGGCAAGAAGTTGATTATATGCCTCAG3’, downstream 5’GTAGATTCCAATGCAGAGATCA3’). An endonuclease Bsu36I recognition site was created by the *A-G* transition. Then the PCR product of 364 bp was digested at 37 °C by Bsu36I restriction enzyme (Promega, USA) overnight and assayed on 2.8% agarose gel stained with SYBR Safe DNA Gel Stain (Invitrogen, USA). DNA sequencing of PCR–RFLP products was used to confirm the results obtained by RFLP.

### PCR-RFLP analysis of the N680S variant

The N680S variant in the exon 10 of FSHR gene was amplified by PCR-RFLP using a set of primers (upstream 5’TTTGTGGTCATCTGTGGCTGC3’, downstream 5’CAAAGGCAAGACTGAATTATCATT3’). A 519 bp DNA fragment was amplified with 35 cycles of 95°C for 30 seconds, 60°C for 30 seconds, and 72°C for 45 seconds. The PCR-RFLP fragment was digested by BsrI (Promega, USA) and 2.8% agarose gel electrophoresis with SYBR Safe DNA Gel Stain. DNA sequencing of PCR–RFLP products was used to conﬁrm the results obtained by RFLP.

### Statistical analysis

The statistical tests were performed using the SPSS 19 (Statistical Package for the Social Science—SPSS Inc., USA). The observed genotype frequencies in patients compared with the expected frequencies under Hardy–Weinberg equilibrium were evaluated by the Chi-square test. ANOVA was used to analyze the differences in age, BMI and basal hormone levels. ANCOVA was used to analyze the differences in other clinical parameters between the study groups. Chi-square analysis was used to analyze the data of infertility etiology, ovarian response, OHSS and pregnancy rate. The haplotype analysis for FSHR was performed using PHASE 2.1. Linkage disequilibrium between SNPs was estimated using Haploview version 3.2 [28]. P<0.05 was considered statistical significance.

## Results

### Frequency of the variants of the FSHR gene

**Figure 1:**
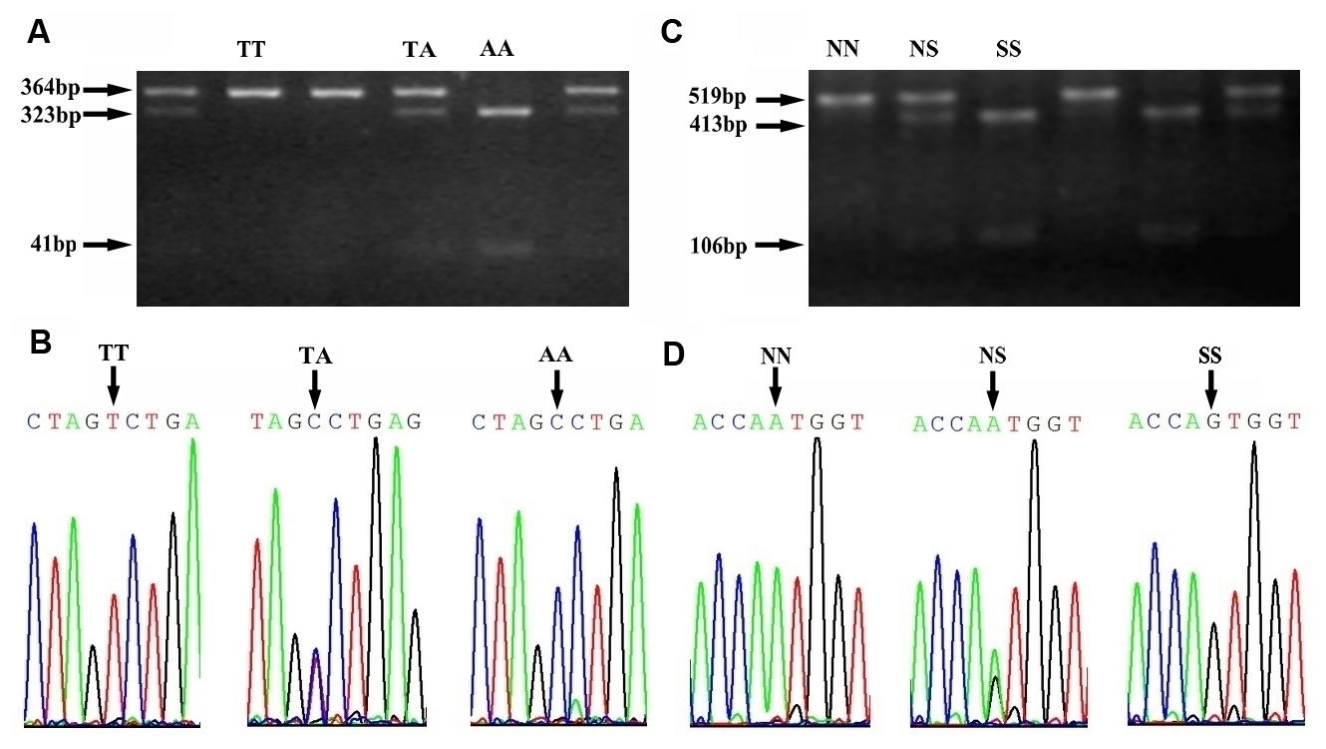
RFLP patterns and DNA sequencing results of SNP Thr307Ala and Ser680Asn. (A) RFLP patterns of SNP Thr307Ala visualized on 2.8% agarose gel. (B) Confirmation of RFLP by DNA sequencing. The arrows indicated the polymorphism at position 307. (C) RFLP patterns of SNP Ser680Asn visualized on 2.8% agarose gel. (D) Confirmation of RFLP by DNA sequencing. The arrows indicated the polymorphism at position 680.

We first established a method to analyze the genotypes of FSHR by RFLP assay. For T307A variant, three different patterns of restriction enzyme digestion were revealed (Figure 1. A). A 364 bp undigested fragment indicated the homozygosity for Thr/Thr (TT), while the digested fragments indicated the homozygosity for Ala/Ala (AA) that resulted in 323 bp and 41 bp fragments. The presence of all three fragments indicated the heterozygote for Thr/Ala (TA). For N680S variant, three different patterns were also shown as in Figure 1.C, where undigested fragment (519 bp) indicated the homozygosity for Asn/Asn (NN); the digested fragments of 413 bp and 106 bp indicated the homozygosity for Ser/Ser (SS); the presence of all three fragments indicated the heterozygote Asn/Ser (NS). PCR products were sequenced to test the validity of the methodology (Figure 1. B and D).

No significant differences were observed between the genotype distribution of the polymorphism and that expected from Hardy–Weinberg equilibrium in patients for both SNPs. PCR-RFLP assay was used to analyze 450 samples from subjects undergoing ART program. As shown in Table 1, the frequencies of FSHR genotypes at position 307 were: Thr/Thr 44.4% (200/450), Thr/Ala 45.6% (205/450) and Ala/Ala 10.0% (45/450). The frequencies for polymorphism at position 680 were Asn/Asn 46.9% (211/450), Asn/Ser 43.8% (197/450), and Ser/Ser 9.3% (42/450). Four haplotypes were eventually obtained, of which, the main AA and GG haplotype frequencies were 66.2% and 30.2%, respectively. The frequencies of the GA and AG haplotypes were 2.6% and 1.0%, respectively.

**Table 1:** Genotyping of FSHR for SNPs 307 and 680 in 450 patients.

**SNP**	**Allele frequency (%)**		**Genotype frequency (%)**	
Thr307Ala	A (T)	67.22	AA (TT)	44.4
	G (A)	32.78	AG (TA)	45.6
			GG (AA)	10.0
Ser680Asn	A (N)	68.78	AA (NN)	46.9
	G (S)	31.22	AG (NS)	43.8
			GG (SS)	9.3

**Figure 2:**
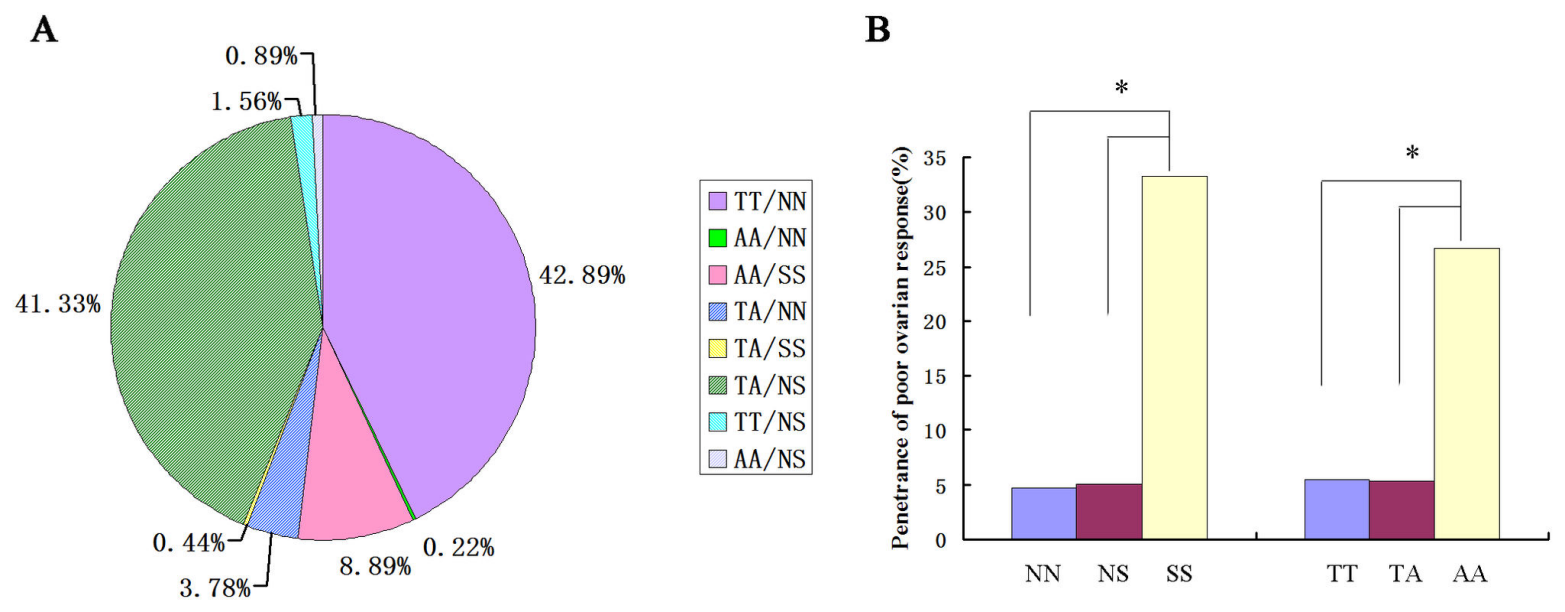
Allele frequency and penetrance of poor ovarian response based on the polymorphism at position 307 and 680 in 450 patients. (A). 450 infertile women undergoing ART program were assessed for the genotypes. Percentage of each allele in 450 women was plotted. (B) In 450 Chinese women who were infertile and recruited in an ART program, the ovarian response was classified to poor (less than five), normal (between five and fourteen) and high (more than fourteen) based on the number of retrieved oocytes. The percentage of the “poor” responders in each genotype was used for the plot. *P=0.000, Chi-square analysis.

To further analyze the allelic distributions, we examined the frequencies of homozygous and heterozygous individuals for SNPs at 307 and 680 (Figure 2. A). The Thr307/Asn680 (TN) and the Ala307/Ser680 (AS) were two most frequently alleles observed in the Chinese women, and 41.33% of women were heterozygous at both alleles. The TN allelic variant had the highest frequency at about 82.48% (193/234) of homozygous individuals. Two other variants Ala307/Asn680 (AN) and Thr307/Ser680 (TS) had much less frequencies, only found in few patients. Both homozygosity and heterozygosity were included to caculate an association between the positions 307 and 680. A significant association was detected between the genotypes at position 307 and at position 680(D’=0.95, r^2^=0.84). The results were discordant in only 31 patients out of all 450 research subjects. 

### FSHR polymorphism and clinical parameters

Clinical and endocrinologic parameters were analyzed based on the polymorphism at position 307 (Table 2) and 680 (Table 3) in the subjects undergoing ART program. The amount of FSH used for the patients was variable from 675 IU to 5400 IU in order to reach a successful stimulation. The basal FSH levels at the position 307 were signiﬁcantly higher in the Ala/Ala group (7.38 ± 2.07mIU/ml) than the Thr/Thr group (6.34 ± 1.75 mIU/ml) or the Thr/Ala group (6.63 ± 1.94 mIU/ml) on day 3 of the menstrual cycle [ P<0.05, 95% CI (6.75, 8.01)] (Table 2). Similarly, the basal FSH levels were statistically and significantly different for the polymorphism at position 680. The basal FSH levels was signiﬁcantly higher in the Ser/Ser group (7.51 ± 2.01 mIU/ml) than the Asn/Asn group (6.31 ± 1.75mIU/ml) or the Asn/Ser group (6.66 ± 1.96 mIU/ml) [P<0.05, 95% CI (6.88, 8.15)] (Table 3). Moreover, there is a correlation between the days for stimulation and the FSHR genotypes. As shown in Table 2 and 3, The Ala/Ala [P=0.001, (position 307)] and Ser/Ser [P=0.009, (position 680)] group required slightly longer stimulation than other groups.

**Table 2:** Clinical and endocrinologic parameters of the subjects undergoing ART program based on polymorphism at position 307.

**Clinical parameter**		**TT (n=200)**	**TA (n=205)**	**AA (n=45)**
Age (years)		31.30±5.02	31.98±5.09	33.16±4.76
BMI (kg/m^2^)		21.60±2.90	21.49±2.76	21.73±2.75
infertility etiology				
Tubal		118	115	20
	Male	6	6	0
	Mix	74	81	23
	Unexplained	2	3	2
Amount of the FSH required for ovulation induction (IU)		2110.37±712.43	2236.33±796.87	2368.06±549.84
Basal FSH levels (mIU/ml)		6.34±1.75^a^	6.63±1.94^b^	7.38±2.07^a,b^
Basal estradiol levels (pmol/l)		44.38±22.65	45.75±39.98	39.07±15.68
Basal LH levels (mIU/ml)		4.78±2.89	4.96±2.73	5.34±2.56
FSH level after down regulation (IU/L)		4.00±2.31	4.00±2.17	4.27±3.16
Estradiol level after down regulation (pg/ml)		24.80±26.05	22.95±31.18	17.01±10.70
LH level after down regulation (IU/L)		2.27±1.70	2.34±1.65	2.82±3.07
Estradiol levels on the day of hCG administration (pg/ml)		4024.02±2432.12	4002.72±2181.05	3939.49±2487.81
LH levels on the day of hCG administration (pg/ml)		1.48±0.79	1.77±1.89	1.55±0.93
Progesterone on the day of hCG administration (ng/ml)		0.99±2.34	0.94±0.70	0.95±0.51
Stimulation length (days)*		11.32±2.15	12.02±2.44	12.62±2.92
Antral follicle count		11.19±5.92	11.41±5.87	11.73±5.86
No. of follicle (d≥14mm) on the day of hCG administration		8.90±3.15	9.29±3.14	8.56±3.31
No. of preovulatory follicles		9.57±3.21	10.01±2.93	9.36±3.00
No. of oocytes		13.10±6.85	13.00±6.14	11.98±7.26

Note: Values are mean±SEM. ANOVA was used to analyze the differences in age, BMI and basal hormone levels. Chi-square analysis was used to analyze the data of infertility etiology. ANCOVA test was used for analysis of other variance (* Values signiﬁcantly different among three groups). P<0.05 was considered as the statistical signiﬁcance. ART =assisted reproductive technology.

^a^ Values signiﬁcantly different among TT and AA groups.

^b^ Values signiﬁcantly different among TA and AA groups.

**Table 3:** Clinical and endocrinologic parameters of the subjects undergoing ART program based on polymorphism at position 680.

**Clinical parameter**		**NN (n=211)**	**NS (n=197)**	**SS (n=42)**
Age (years)		31.36±4.97	31.96±5.19	33.17±4.51
BMI (kg/m^2^)		21.52±2.84	21.61±2.83	21.58±2.74
infertility etiology				
	Tubal	119	114	20
	Male	6	6	0
	Mix	84	74	20
	Unexplained	2	3	2
Amount of the FSH required for ovulation induction (IU)		2107.34±711.70	2251.31±802.01	2355.06±522.76
Basal FSH levels (mIU/ml)		6.31±1.75^a^	6.66±1.96^b^	7.51±2.01^a,b^
Basal estradiol levels (pmol/l)		44.75±23.26	43.57±33.38	47.64±52.12
Basal LH levels (mIU/ml)		4.79±2.82	4.93±2.78	5.51±2.61
FSH level after down regulation (IU/L)		4.04±2.32	3.98±2.14	4.23±3.24
Estradiol level after down regulation (pg/ml)		24.84±25.56	22.70±31.60	17.15±11.10
LH level after down regulation (IU/L)		2.35±1.78	2.30±1.62	2.68±3.03
Estradiol levels on the day of hCG administration (pg/ml)		4062.71±2430.11	3997.84±2199.95	3754.20±2356.27
LH levels on the day of hCG administration (pg/ml)		1.50±0.83	1.75±1.91	1.56±0.94
Progesterone on the day of hCG administration (ng/ml)		0.99±2.28	0.94±0.70	0.92±0.54
Stimulation length (days)*		11.38±2.16	12.10±2.57	12.21±2.56
Antral follicle count		11.32±6.02	11.37±5.88	11.36±5.26
No.of follicle (d≥14mm) on the day of hCG administration		9.03±3.31	9.24±2.96	8.19±3.29
No. of preovulatory follicles		9.70±3.34	9.96±2.74	8.98±3.01
No. of oocytes		13.07±6.76	13.20±6.17	11.12±7.29

Note: Values are mean±SEM. ANOVA was used to analyze the differences in age, BMI and basal hormone levels. Chi-square analysis was used to analyze the data of infertility etiology. ANCOVA test was used for analysis of other variance (* Values signiﬁcantly different among three groups). P<0.05 was considered as statistical signiﬁcance. ART =assisted reproductive technology.

^a^ Values signiﬁcantly different among NN and SS groups.

^b^ Values signiﬁcantly different among NS and SS groups.

However, no statistically signiﬁcant difference was observed among three groups in these parameters: age, BMI, infertility etiology, basal estradiol and LH levels, amount of the FSH required for ovulation induction, FSH, estradiol ,LH levels after down regulation, estradiol, LH , progesterone levels, number of antral follicle and follicle (d≥14mm) on the day of hCG administration, preovulatory follicles, and number of retrieved oocytes (Table 2 and 3 ).

### FSHR polymorphism and the ovarian response

The ultrasonographic parameters showed a potential association between FSHR polymorphisms and the ovarian response in subjects undergoing ovarian induction. The ovarian response of different FSHR gene polymorphisms for the residues 680 during ovulation stimulation was shown in Figure 2. B. It is interesting that women with the homozygous for Ser/Ser had signiﬁcantly higher rates of poor response (Chi-square analysis:P=0.000). Also, we observed the similar effect for residue 307 that women with the homozygosity for Ala/Ala had signiﬁcantly higher rates of poor response (Chi-square analysis:P=0.000).

### FSHR polymorphism and OHSS

OHSS occurred in 54 women in the study group of 450 subjects prospectively enrolled in the ART program. Chi-square test revealed that there was no signiﬁcant association between the occurrence of OHSS and the genotypes at either position 307 or 680: Thr307Ala (P=0.254), Asn680Ser (P=0.092). Also, the biochemical pregnancy rates and clinical pregnancy rates were not significantly different among three polymorphisms at positions 307 or 680.

## Discussion

It has been previously reported that the response of ovaries to FSH stimulation is variable in ART program, and FSHR polymorphisms of Thr307Ala and Asn680Ser may be related to the response of the patients to FSH stimulation. One study suggested that the effects of the FSHR polymorphism are independent of ethnic background, in spite of lack of the data from Chinese woman population [29]. To verify this, we, for the first time, analyzed the association of FSHR polymorphisms with the ovarian response in relatively large Chinese population (n=450). We investigated the association of FSHR polymorphisms with clinical and endocrinologic parameters in Chinese women. Present study revealed that the distribution frequencies of the two polymorphisms in Chinese women are consistent with other ethnic groups [12,21,30].. Interestingly, the stimulation for Ala/Ala and Ser/Ser was slightly longer than others in Chinese women, which were not seen in other reports. We speculate that ethnicity or stimulation protocol may attribute to this difference.

It has been reported that subjects with homozygous Ser/Ser have higher basal levels of FSH, and require more exogenous FSH to induce ovulation [4,18,19]. Basal FSH level has been used to estimate the ovarian reserve, in which high basal levels usually predict a higher ovarian threshold to FSH responses. Boudjenah R et al found that subjects with Ser680 variant had much higher FSH level at the day 3 of menstrual cycle, and the amount of administered FSH was similar for all genotypes in French women [31].Our findings confirmed that the Ser/Ser (680) and Ala/Ala (307) are associated with a decreased ovarian reserve, but not with the exogenous FSH for the ovuation stimulation, which confirmed to previously report. However, there seemed to be some controversies to this notion. Achrekar et al failed to detect the association of polymorphism at position 680 with the basal FSH levels and the amount of exogenous FSH, and found that the maximum amount of exogenous FSH was required for the subjects with homozygous Thr/Thr in Indian women [22]. Whether these differences attribute to ethnicity or stimulation protocol still needs to be investigated.

Previous studies suggested that the same dose of FSH resulted in much lower estradiol levels in subjects with homozygous Ser/Ser than in Asn/Asn in German women [32]. In addition, some evidence suggested a positive linkage of estradiol levels to 2p21 in whites, where the FSHR gene is located [33]. However, we did not observe a significant difference of estradiol levels between polymorphisms at 307 or 680 on the day of hCG administration in Chinese women. Interestingly, similar to preexisting studies [18,32], we showed that the estradiol levels were higher in subjects with Asn/Asn allele than Ser/Ser allele, whereas Ala/Ala genotype showed the lowest estradiol levels in these three groups on the day of hCG administration, which was different from the levels in Indian women [22]. However, since only 50 women were recruited in the Indian study, we could not conclude if it is due to the difference of ethnicity or sample size.

It has previously been reported that the Ser/Ser carriers may have an increased risk of poor outcome during COH [34]. In the present study, more women with Ala/Ala and Ser/Ser poorly responded to FSH. However, the presence of Thr/Thr and Asn/Asn among poor responders indicated that Ala/Ala and Ser/Ser may not be the only cause of poor response to FSH. Other genetic and environmental factors may contribute to the low response of FSH in ART.

Although previous study reported that FSHR polymorphism at position 680 could be used to predict the severity of symptoms of OHSS patients, but could not predict the occurrence of OHSS [21]. We did not observe the association of FSHR genotypes with OHSS, a serious potential life-threatening complication in ART. Due to insufficient clinical data, we did not analyze OHSS grade in the present study.

A possible linkage between Thr307Ala and Asn680Ser has been reported in the infertile women. A complete linkage has been reported between polymorphisms Thr307Ala and Asn680Ser of FSHR gene in Japanese women (n=522) [20], while other group did not observe a complete linkage of these two polymorphisms in Brazil women, because 12 out of total 51 fertile women showed discordant results (r=0.6363, p=0.001, n=51) [25]. In the present studies, we also studied the linkage between Thr307Ala and Asn680Ser in the infertile Chinese women. The data demonstrated a nearly complete linkage between two polymorphisms (D’=0.95, r^2^=0.84, n=450). These data, together with others, suggest a use of Thr307Ala and Asn680Ser in FSHR gene as TAG-SNPs for potential genotyping in ART.

In all, the results in the present study provided new additions to the understanding of the impact of FSHR SNPs on exogenous FSH induction. Personalized FSH therapy may be used in ovarian stimulation program according to the patient’s genetic background in clinical settings. In ART treatment, genetic testing may help to identify patient’s likely response when given FSH in relation with genetic polymorphisms, and provide tools for the determination of optimizing drug dose to magnify the clinical benefit of ART.
